# Resource competition promotes tumour expansion in experimentally evolved cancer

**DOI:** 10.1186/s12862-017-1117-6

**Published:** 2017-12-27

**Authors:** Tiffany B. Taylor, Anastasia V. Wass, Louise J. Johnson, Phil Dash

**Affiliations:** 1 School of Biological Sciences, University of Reading, Whiteknights, Reading, RG6 6AH UK; 2 Milner Centre for Evolution and Department of Biology and Biochemistry, University of Bath, Claverton Down Road, Bath, BA2 7AY UK

**Keywords:** Experimental evolution, Dispersal, Metastasis, Resource competition, Microenvironment, Plasticity, Epigenetic

## Abstract

**Background:**

Tumour progression involves a series of phenotypic changes to cancer cells, each of which presents therapeutic targets. Here, using techniques adapted from microbial experimental evolution, we investigate the evolution of tumour spreading - a precursor for metastasis and tissue invasion - in environments with varied resource supply. Evolutionary theory predicts that competition for resources within a population will select for individuals to move away from a natal site (i.e. disperse), facilitating the colonisation of unexploited resources and decreasing competition between kin.

**Results:**

After approximately 100 generations in environments with low resource supply, we find that MCF7 breast cancer spheroids (small in vitro tumours) show increased spreading. Conversely, spreading slows compared to the ancestor where resource supply is high. Common garden experiments confirm that the evolutionary responses differ between selection lines; with lines evolved under low resource supply showing phenotypic plasticity in spheroid spreading rate. These differences in spreading behaviour between selection lines are heritable (stable across multiple generations), and show that the divergently evolved lines differ in their response to resource supply.

**Conclusions:**

We observe dispersal-like behaviour and an increased sensitivity to resource availability in our selection lines, which may be a response to selection, or alternatively may be due to epigenetic changes, provoked by prolonged resource limitation, that have persisted across many cell generations. Different clinical strategies may be needed depending on whether or not tumour progression is due to natural selection. This study highlights the effectiveness of experimental evolution approaches in cancer cell populations and demonstrates how simple model systems might enable us to observe and measure key selective drivers of clinically important traits.

**Electronic supplementary material:**

The online version of this article (10.1186/s12862-017-1117-6) contains supplementary material, which is available to authorized users.

## Background

Solid tumours are largely curable if they are treated before they spread. However, once cancer cells become metastatic and move beyond the location of the primary tumour, mortality rates increase drastically [1]. Metastatic and invasive tumours – those that spread beyond the primary location – show increased spreading to adjacent tissues, which is caused by increased cell motility [2]. As such, targeting pre-metastatic traits might be a novel approach to prevent the evolution of cancerous traits that would facilitate spreading and invasive behaviours [3]. Indeed there is some evidence to suggest that oxygenation of a tumour inhibits metastasis [4]. However, cancer cells do not need to evolve motility systems de novo*,* but can co-opt existing mechanisms enabling rapid changes in phenotype [5]. Motility is a normal cellular behaviour for many human cell types, either constitutively, or under particular conditions such as development and tissue repair. Therefore, to understand the processes underlying changes in the behaviour of cancerous cells we must first understand the drivers of change.

Solid tumours, if left untreated, will often progress to metastatic tumours [6]. This is puzzling from an evolutionary perspective. Unlike other hallmarks of cancer such as apoptosis resistance, evasion of growth suppression, or replicative immortality [7], metastasis is not immediately concerned with cell survival or reproduction and appears to have no inherent selective value within a tumour [8]. Nor does motility ensure cell fitness outside the original tumour: of the estimated 10^6^–10^7^ cells that emigrate daily from a developed neoplasm [9], the vast majority die rather than initiating secondary tumours.

One potential solution to this evolutionary paradox is suggested by an analogy between metastasis and ecological dispersal [10]: an indirect benefit accrues to dispersers if the source population consists of closely related individuals competing for scarce resources [11]. This key prediction was tested in bacterial populations where relatedness between spreading and non-spreading mutants was experimentally manipulated [12]. This study concluded that populations of spreading cells that dispersed further increased distances between competitors and therefore reduced overall cell-cell competition. The consequence being that even under very high costs of dispersal, clonal populations of spreading bacteria were more fit compared to a mixed (low related) population. Therefore, the benefit to moving away from the primary tumour is two-fold: (i) the small proportion of dispersers that successfully colonise a new site will face less competition and reach untapped resources to facilitate rapid growth; and, (ii) by moving away, the cell is reducing competition between its clonemates at the primary tumour site. By increasing the fitness of its clonemates, who will leave more descendants, the disperser is indirectly increasing its own fitness – even if it perishes and fails to establish a metastatic tumour elsewhere [13].

Tumour cells are likely to face exploitation competition in growing neoplasms [14]. Competition will occur for resources including nutrients and oxygen in the early stages of cancer [15], as these can only diffuse approximately 1 mm into a tumour from surrounding blood capillaries alone [16–18]. There is some evidence that these hostile microenvironments favour motility. For example, in uterine cancer [19] and soft tissue sarcomas [20], hypoxia has been shown to be linked with greater likelihood of metastases. Therefore, as the primary tumour grows, resource competition between clonemates is likely to be quickly established. Evolutionary theory predicts that this will drive selection for dispersal.

Natural selection has been detected in clinical tumour samples by applying statistical techniques from population and evolutionary genetics to end-point data [21–23]. However, to gain a deep understanding of the quantitative effect of natural selection in cancer progression we must first go back to evolutionary basics. The power of an experimental evolution approach is that it enables causality of selection to be tested through hypothesis driven experiments. Recent dispute over the importance of natural selection in tumour progression [24, 25] has highlighted the need for a quantitative understanding of the forces leading to cancer progression.

Viewing cancer progression as an evolutionary and ecological process is becoming more common practise; providing new insights into progression and treatment of cancers [26–29]. In particular, dispersal evolutionary simulation models have been utilised to explore the evolution of spreading behaviour revealing the role of metabolism and nutrient competition [30], the microenvironment [31] and resource heterogeneity [32] in driving the evolution of cell migration. A common theme is that the nutrient environment plays a critical role in selecting for increased cell motility, which provided the context for this study.

The next step is to experimentally validate these predictions for which we must develop experimental techniques that can accurately measure the effect of key selective drivers on the evolution of clinically relevant traits in tumour cell populations. In Taylor et al. [33], we advocated adapting the techniques of experimental evolution in microbes to cancer research (see also [34]). Here, we report the findings of the experiment we proposed to determine the role of cell-cell competition in causing increased spheroid spread, which models an early stage of metastasis seen within primary tumours (for a discussion of the advantages and limitations of spheroids spread as a model for metastasis, see [35]).

## Methods

### Aim

The aim of this study was to identify resource supply (high or low) as a driver for the evolution of spheroid spreading in a population of breast cancer cells. Six independent selection lines of MCF7 breast cancer cells were established; 3 replicate lines were maintained under low resource supply and 3 under high resource supply. Every 7 days, after the cells had become confluent, 10% were transferred to fresh media. Transfers were made each week for 12 weeks. Comparisons between lines, and with the ancestor, gives a measure of the effect of selection over time on cell phenotype.

### Cell culture

Experiments were performed using MCF7 cells (ATCC® HTB-22™; passage number 17) [36], a relatively slow moving, non-metastatic cell line (although derived from a metastatic site). Cells were grown as monolayers in 25 cm^2^ tissue culture flasks with non-phenol red Dulbecco’s Modified Eagles Medium (DMEM) containing 0.5% or 5% (depending on treatment group) foetal bovine serum (FBS), 1% Penicillin, 1% streptomycin, and 2 mM L-glutamine. Incubated at 5% CO_2,_ 37 °C.

### Selection lines

MCF7 cell lines were maintained in low (0.5% FBS) or high (5% FBS) resources for 12 weeks. MCF7 cells have a generation time of approximately 24 h. Three independent replicate lines were maintained within each treatment group. Every 7 days a random subpopulation of 1000 - 4000 cells were transferred to fresh medium. 7 days were sufficient to allow cells to cover the base of the cell tissue flask, forcing cells to compete for space and resources.

Cells were removed from the incubator and the old media was discarded. To detach, cells were washed in 5 ml Phosphate Buffered Saline (PBS), treated with 2 ml Trypsin-EDTA and incubated at 37 °C for approximately 5 min. Cells were re-suspended in DMEM and 10% were transferred to fresh media. At each transfer a sample of each cell line was frozen down to allow resurrection for further post hoc phenotypic analysis. Cells were passaged as normal and the remaining solution was centrifuged at 1000 rpm for 3 min. The supernatant was discarded, cells re-suspended in freezing buffer (10% dimethylsulfoxide (DMSO) and 90% FBS) and stored at −80 °C. After 24 h, the vials were moved and placed in liquid nitrogen.

### Spheroid spread assays

Spheroids offer a tangible in vitro model that more accurately reflect clinical expression profiles compared to monolayer cultures [35, 37], and have been previously used to study spreading behaviour of cancers (e.g. [38–40]). Cells from the selection lines were grown in non-adhesive flasks for 24 h, allowing them to form spheroids (small in vitro tumours), roughly spherical clusters of approximately 1000 cells. To avoid any initial responses to change in media, spheroids were cultured in the same media that they were to be tested in. When returned to flasks with a suitable surface, spheroids will adhere and the constituent cells will move outwards, eventually forming a monolayer. We allowed spheroids to adhere for 4 h to the surface of 12-well tissue culture plates, and calculated the areas covered by cells dispersing from the spheroid by analysing images taken on a Zeiss A1 Inverted Epifluorescent microscope using Nikon NIS Elements and analysed with ImageJ software [41]. Six spheroids were measured within each well and 3 independent wells were measured. Spheroids within wells were randomly paired between photos taken at time zero and 72 h and the difference calculated. We were unable to measure the same spheroids between time points as magnification adjustment and manual tracking was necessary to accommodate rapid spread to keep spheroids within the field of view.

### Growth rate assays

Cells were transferred to 6-well plates and seeded at 2% confluency. The number of cells present were counted using a haemocytometer at time 0 and after 72 h to gain an initial cell count prior to cell adherence and total end cell count per well, respectively. Images were taken at 12 h intervals at the same locations within each well, and cells counted using ImageJ. The average for 3 independent replicates across all 3 cell lines was taken. Growth rate was calculated using the formula:

$$ \frac{\ln \left(\frac{N_2}{N_1}\right)}{\left({t}_2-{t}_1\right)} $$

where t_2_ is the time at the end of the experiment (72 h), t_1_ is the time at the beginning of the experiment (12 h), N_2_ is the number of cells present at t_2_ and N_1_ is the number of cells present at t_1_. The first image was taken at 12 h to allow cells time at adhere to the well surface. Between 12 h and 72 h growth rate is assumed to be exponential as cells have not yet reached confluency.

### Timelapse cell motility assay

Cells from the selection lines were seeded in a 12-well plate at approximately 5000 cells/well. Each cell line was cultured in the media it had been adapted to and each well had 5 points chosen at random from which to observe the cells. A Nikon TE200 Timelapse System with NIS Elements 3 was used to capture a bright-field image at the points chosen every 15 min for just over 48 h (actual, 52 h and 15-min). These images were collated to form a timelapse video allowing the individual cells within the field of view to be tracked using ImageJ and MtrackJ. This tracking allows observation of whether or not a cell divides and can be used to calculate the speed of individual cells. Speed was calculated as the total length (in microns) moved by the cell divided by the time (in hours) the cell was tracked for. As many cells as possible were measured and this was repeated for 3 independent replicates. In addition, distance to point was also measured using tracking data. Here, the distance a cell moves between two time-frames (every 15 min) is recorded against time. The benefit of this measurement is that it allows the proportion of cells across a population that are moving at a particular time point to be calculated, rather than tracking the motility of an individual cell. This, combined with the cell speed data, gives an indication of population behaviour across time.

### Statistics

Analyses and figures were produced on IBM® SPSS® Statistics 24.0. Significance of treatment (high or low resource supply) on phenotype was analysed parametrically using general linear models (GLMs). Terms used in the model are defined as the following: ‘Spheroid area’ [response variable], average area covered by spheroid spread after 72 h. Areas were square-root transformed to correct for right skew that is typical of area data, and divided by growth rate to correct for expansion of spheroid driven by differences in growth rate rather than spreading behaviour; ‘Experimental media’ [explanatory variable, factor], high or low resource supply in experimental conditions; ‘Evolved environment’ [explanatory variable, factor], ancestor and high or low resource supply maintained in evolving lines; ‘Cell speed’ [response variable], the average distance (429 Ancestor, 298 Evolved high-resource supply and 356 Evolved low-resource supply) the cells moved in micrometres per hour over a 52-h period. A Kruskal-Wallis and skew test were used to measure the distribution of ‘distance to point’ data. In each case the effect of the well within the tissue culture plate was measured. This was to ensure there were no microenvironment differences between wells – this was non-significant in all cases and therefore removed from the final model. Replicate is treated as a random factor. Tukey tests were performed between treatment groups within cell line. In all cases the area dispersed was square-root transformed.

## Results

**Fig. 1:**
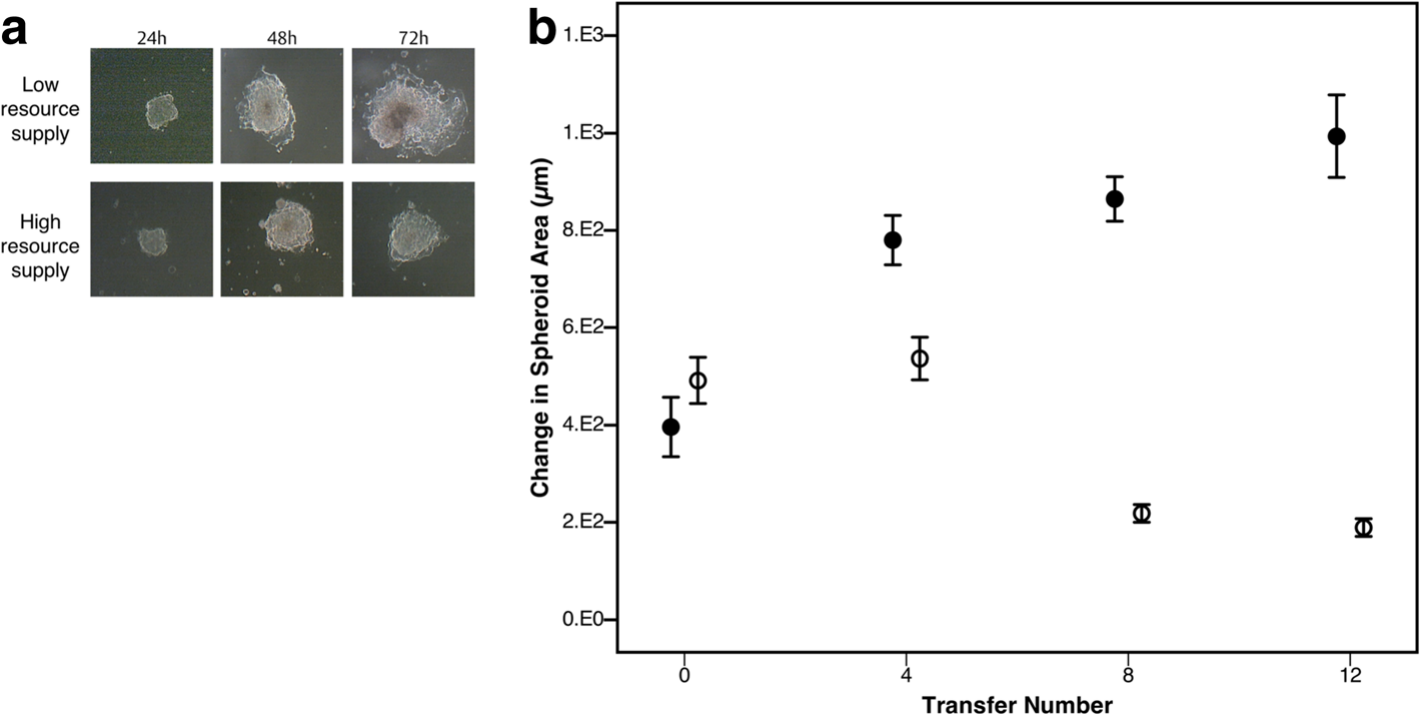
Spheroid spread over time. **a**. Images of MCF7 cell populations demonstrating differences in spheroid (in vitro tumours of approximately 1000 cells) spread over a 72-h period. Cell motility was calculated by measuring the change in surface area covered by spheroids over time; **b** Average spheroid spread (across 3 replicate lines) for selection lines under high and low resource supply over 12 weekly transfers. Spreading was measured 72 h after adhesion of the spheroid, and in the selective environment for each line (i.e. the motility of the low resource supply line was measured in low resource medium). Closed circles represent lines evolved under low resources, open circles are lines evolved with access to high resources. Areas are square-root transformed (± 1 SE)

Images of cells across different transfer points were taken over 72 h to measure the distance of spread of spheroids (clumps of around 1000 self-adhered cells; Fig. 1a) from an initial adhesion site. The change in area over time was used as a measure of spheroid spread. At transfer 0, there was no difference between spheroid spread in the different treatments for resource supply (F_1,9_ = 1.426; *P* = 0.263). Furthermore, there was no significant difference between initial spheroid size between selection lines (F_2,101_ = 0.163; *P* = 0.848). However, over time we find resource supply has a significant effect on spreading (F_1,105_ = 103.61; *P* < 0.001), with faster spreading emerging in lines maintained under low resource supply (F_3,105_ = 23.872; P < 0.001) (Fig. 1b). These effects are unlikely to be driven by the microenvironment as there was no significant difference between spreading distances of replicate spheroids between wells (F_2,89_ = 1.423; *P* = 0.246).

**Fig. 2:**
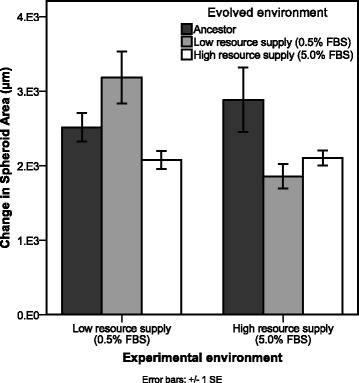
Common garden experiment. Average change in spheroid area (square-rooted) over 72 h for ancestor and evolved high and low resource selection lines in high and low resource environments. Areas are square-root transformed (± 1 SE)

After 12 transfers, spheroids from ancestor and both selection lines were allowed to grow and spread under high and low resource conditions (a “common garden” experiment in ecology). The ancestor showed no difference in spreading area under high or low resources supply (F_1,4_ = 0.613; *P* = 0.477). However, the evolved lines were found to respond differently; high-resource selection lines showed no difference in motility between the two resource conditions, but low-resource selection lines showed slower motility in high-resource medium compared to low-resource medium (Fig. 2; F_1,8_ = 10.502; *P* = 0.012).

**Fig. 3:**
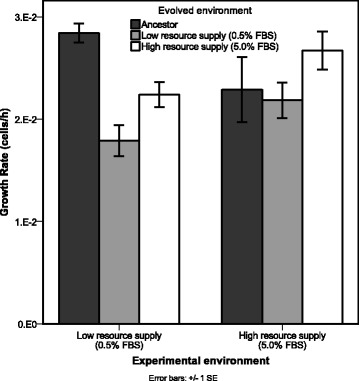
Growth rate of ancestral and evolved lines. Average growth rate, represented as the increase in cell number per hour (± 1 SE). Growth rates of ancestral (dark grey bars), low resource supply evolved lines (light grey bars), and high resource supply evolved lines (white bars) are shown when grown in high resources (5.0% FBS) and low resources (0.5% FBS)

To correct for differences in spheroid spread that might be due to growth, spreading area is divided by growth rate (Fig. 3). We found that growth rates of selection lines and ancestor were not different with access to high resource supply (5.0% FBS) (F_2,6_ = 1.171; *P* = 0.372), however under low resource supply (0.5% FBS) we find significant differences between ancestral and evolved lines (F_2,6_ = 17.601; *P* = 0.003). When grown in 0.5% FBS (low-resource supply), lines evolved under high and low resource supply showed lower growth rates than the ancestor, despite the low-resource evolved lines showing higher spheroid spreading (Tukey: Ancestor v. 5.0% FBS *P* = 0.034; Ancestor v. 0.5% FBS, P = 0.003). This suggests that spreading behaviour is not explained by growth.

**Fig. 4:**
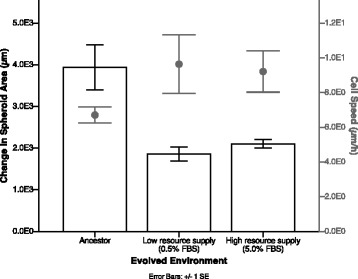
Spheroid spread versus cell speed. Bars represent the average change in spheroid area over 72 h in high (5% FBS) resource media for ancestor and lines evolved in low and high resources (± 1 SE). Closed circles represent average cell speed of cells in high resource media for ancestor and evolved lines. Areas are square-root transformed (± 1 SE)

The speed of cell movement was measured using video tracking of motile cells. Individual cell motility in lines evolved in high and low resources were not significantly different from the ancestor, or each other (Fig. 4; Tukey test: 0.5% v. 5.0%, *P* = 0.968; 0.5% v. Ancestor, *P* = 0.282; 5.0% v. Ancestor, *P* = 0.377). In addition, we calculated the ‘distance to point’, which measures the distance a cell moves between time frames (Additional file 1: Figure S1). Populations evolved in a low resource supply show fewer non-motile cells at a given time point compared to those evolved with high resource supply and ancestral lines. In addition, the distribution of ‘distance to point’ values across different lines is not equal (Kruskal-Wallis, *P* < 0.05). Ancestors show the highest positive skew (4.164 ± 0.011) followed by lines evolved in high resource supply (3.176 ± 0.015) and lines evolved in low resource supply (2.485 ± 0.014). Finally, these trends hold true across time, when distance to point is measured after 15 min, 24 h and 48 h. Together, these data suggest that in low resource supply lines: there are fewer non-motile cells; a greater proportion of cells are spreading further between time points; and, these differences are sustained across time. This would mean that cells evolved in low resource supply would spread more quickly, as distances between cells increases, compared to ancestral and high resources supply lines.

## Discussion

We grew independent cancer cell populations over multiple generations to measure the effect of resource supply on spheroid spread. We find a significant difference in spreading area over time driven by resource supply. In particular, as predicted by dispersal evolutionary theory, when resources are scarce (driving competition between cells) spheroids are under selection to spread further compared to when they have access to a high resource supply in the same time period (Fig. 1b). Despite the energetic demands of cell motility, spheroids in high resource environments show reduced spreading, and spheroids with access to low resource supply evolve increased spreading. In addition, a common garden experiment – where evolved lines are grown in both low and high resource environments – reveals that cell populations evolved with a low resource supply show phenotypic plasticity in spheroid spread, such that the rate of spread depends on their current nutrient environment, inducing faster spreading in low resources supply compared to the same population in high resource conditions (Fig. 2). Cell populations evolved with access to a high resource supply do not respond to changes in resource environment.

These results are consistent with competition selection as a driver of dispersal; as 40 generations is a relatively short timeframe, this would most likely be selection on standing genetic variation, which can be substantial in MCF7 due to genome instability [42]. However, the complexity of human gene regulatory pathways and the known sensitivity of cancer cell lines to subtle changes in environmental conditions [43], as well as uncharacterised experimental effects [44] necessitates caution in interpretation. Epigenetic responses to the environment that persist across cell generations could also cause stable changes in phenotype such as are seen here. Distinguishing between these alternative explanations should be a high research priority, not only out of academic interest in the precise level of adaptationism appropriate to cancer biology, but also because the difference may have clinical implications. If metastasis evolves as the result of genetic changes, effective preventative treatments might be those that focus on keeping the cancer cell effective population size and mutation rate low; however, if metastasis emerges as a response to environment, it is of greater importance to understand and control tumour microenvironments.

Hostile microenvironments may both directly cause and selectively favour tumour spread [45], and could pose particular danger in promoting metastasis. Human tumour cells contain the whole human genome, and so are capable of phenotypic change via complex physiological, epigenetic and developmental responses in addition to evolutionary response to natural selection [46]. Models from Waclaw [47] suggest that even short-range cellular migratory activity can markedly increase the rate of tumour growth (i.e. fitness of the tumour cells), even in the absence of changes in cellular growth rates. We found that measurable, stable changes in spheroid spreading behaviour occurred quickly (within 4 weeks), suggesting this trait could evolve rapidly in vivo*.* Although, care must be taken when translating findings from the lab to in vivo*,* as the selective environments will greatly differ.

A cell’s microenvironment is also a product of the population in which it resides. Within this experimental setup, we are considering motility at the level of the population (within a spheroid) rather than the individual (a cell). In other words, we are looking at the effect of resource supply in determining the spread of a tumour rather than individual cancer cells. We found spheroids that were evolved with access to a high resource supply showed reduced spread over time (Fig. 1b). This pattern was not driven by changes in cell motility, as no significant differences in cell motility were seen over time (Fig. 4). This suggests that access to resources during evolution is determining the patterns of spheroid spread independently of cell motility. One interpretation might be that cells evolved in starvation, rather than generally getting faster, are more fit if they move further from cells around them to reduce cell-cell competition – a key prediction in dispersal evolution [10]. Our data finds support for this hypothesis; we find that cells evolved under low-resource supply move consistently across time, and the distance they move slightly increases. In comparison, cells evolved in high-resource supply move the same distance between time points. This suggests that cells evolved in low-resource supply are moving further from the cells around them. The consequence for spheroid spread would be that cells towards the centre of the spheroid mass would have more space to move into if distances between cells at the periphery were greater, increasing spheroid spreading rate.

Dispersal plays a crucial role in a range of evolutionary and ecological processes and as such there has been a major effort to understand its evolution. A central factor that has been highlighted both theoretically [11, 48–50] and empirically [12] is that dispersal is likely to be favoured by selection if it reduces kin competition (here, competition between clonemates). These studies find that even under extremely high costs, dispersal is still favoured when populations are clonal due to the indirect fitness benefits gained from reducing competition between clonemates left in the natal patch. This is because these clonemates will pass on genes shared by the dispersing cell, even if the dispersing cell does not survive to do so itself.

Dispersal theory has previously been applied to cancers to try to predict the impact of the microenvironment on the emergence of motility and metastasis in cancer cell populations [30–32]. However, in these cases the evolution of dispersal was only considered from the perspective of individual cell fitness. Although these studies concur that competitive environments (low nutrient or hypoxic) select for increased tumour spread, they do not consider the role of cell-cell (kin) competition as a driver for the evolution of cell dispersal. Considering the inclusive fitness of the cell – that is, taking into account not only its own reproductive success, but its effects on its relatives – may help solve the paradox as to why metastasis evolves despite high mortality rates of metastasising cells.

Dispersal can therefore be considered as a social behaviour. Social evolution – evolution under the consideration of inclusive fitness – is an area that has been extensively studied using experimental evolution and provides interesting opportunities for further study in cancers [51]. Cell behaviour is likely to be dependent on the social context (i.e. cellular behaviour changes when acting as an individual cell compared to within a tumour) [52, 53]. Our results find that individual cell speed and spheroid spread are not aligned, possibly because the social context is different between the experimental setups: cell speed is measured in a 2D monolayer culture, whereas spheroid spread starts as a 3D multi-cell aggregate. Incorporating social evolution into cancer evolution is likely to reveal new insights into the levels of selection across tumours and bridge gaps in our understanding of the evolution of multicellularity. Recent work has shown that even within a tumour, heterogeneity is established early and as such, evolutionary trajectories could be different across a very small scale [54–56]. In this study, however, while we do not measure a significant difference in cell speed, we do find that there are differences in the distance travelled between time frames between selection lines within a monolayer culture. This suggests that selection is having a measurable effect on the phenotype of cell motility within a 2D environment, as motility is a function of both speed and frequency of cell movement. Moreover, this suggests that selective pressures in one environmental structure (2D) can have important clinical consequences in alternative environments (3D) that are not clear until measured.

It is important to acknowledge that we do not claim to replicate in vivo conditions within our experimental design, in fact it is our aim to simplify the environment as much as possible. As such, there will be key differences, such as replicating cell behaviours from a structured 3D tumour to 2D in vitro assays. However, by simplifying experiments into a 2D environment we can develop methods that are easy to repeat and measure. Simplicity of design is a major strength of experimental evolution as it captures the influence of isolated selective drivers in the absence of biological noise – thus improving overall generality of results. Spheroids offer an ideal in vitro model for studying tumour spreading. Spheroids are a very simple 3D culture; it allows cells to form cell-cell adhesions and then spread. This enables us to see whether cells change their behaviour between a 2D and 3D environment.

Translation of results from experimental cancer evolution studies into potential preventative or therapeutic approaches to cancer treatment is not a trivial task. A hypothesis driven approach, as is seen in experimental evolution studies, can only highlight key selective drivers of clinically relevant cancerous traits in the absence of in vivo noise. As this field develops this information, in combination with front-line research from cancer biologists and clinicians, will reveal novel treatment strategies – such as prevention approaches in patients with high-risk of cancer prior to tumour detection – and confer greater understanding and predictive power to the evolution of clinically relevant traits such as metastasis and drug resistance.

## Conclusions

Cancer researchers face a daunting challenge – to harness evolutionary theory in a clinically meaningful way, and further our understanding of the progression of cancers. To meet this challenge, we must design hypothesis-driven experimental systems to effectively test theoretical predictions of cancer evolution [33, 34]. An experimental evolution approach can help systematically address central questions such as: what is the balance between ecological and evolutionary processes? Can we distinguish between genetic and epigenetic evolutionary changes? And, can we repeat the same evolutionary patterns across different environments and across different cancers? The next step would be to complement experimental evolution data with genomics and transcriptomics to allow changes in coding and regulatory regions to align with phenotypic changes over evolutionary time – giving a clearer indication of how genotype maps with phenotype.

This study uses experimental evolution to observe, in real time, the evolution of a key cancer trait and precursor to metastasis – tumour spreading. We find that low resource supply drives the evolution of spheroid spread. This result aligns with predictions from dispersal evolutionary theory. Seminal experimental evolution studies with microbes have fundamentally changed our understanding of evolution (for review see [57]) and there is strong potential for similar advancements in cancer biology. However, this task is not as simple as repeating existing experiments in a new system. While cancer cell populations share many similarities with microbes that make them amenable to experimental evolution studies [33, 34, 54], they also present many new challenges. There is much greater potential for both stable and transient epigenetic effects on phenotype, and factors such as cancer types and genetic backgrounds will introduce further complexity. However, this study introduces a promising starting point for the development of experimental techniques to detect, measure and quantify key evolutionary processes in cancers.

## Additional file

Additional file 1:
**Figure S1.** Histogram showing distribution of distance to point measurements after 0.25, 24 and 48 h. Blue bars represent ancestral populations, green bars represent populations evolved in low resource supply (0.5% FBS) and pink bars represent populations evolved in high resource supply (5.0% FBS). (PDF 5 kb)

## Abbreviations

DMEM: Dulbecco’s modified eagles medium
DMSO: Dimethylsulfoxide
FBS: Foetal bovine serum
MCF-7: Michigan cancer foundation-7
PBS: Phosphate buffered saline
UR: University of Reading, Reading, UK
US: University of Sheffield, Sheffield, UK
