# Supplementary figures and images for: Resource competition promotes tumour expansion in experimentally evolved cancer
Source: BMC Evol Biol. 2017 Dec 27;17:268. doi: 10.1186/s12862-017-1117-6 (PMC5745887; doi:10.1186/s12862-017-1117-6)

Evolved environment

- Ancestor
- Low resource supply (0.5% FBS)
- High resource supply (5.0% FBS)

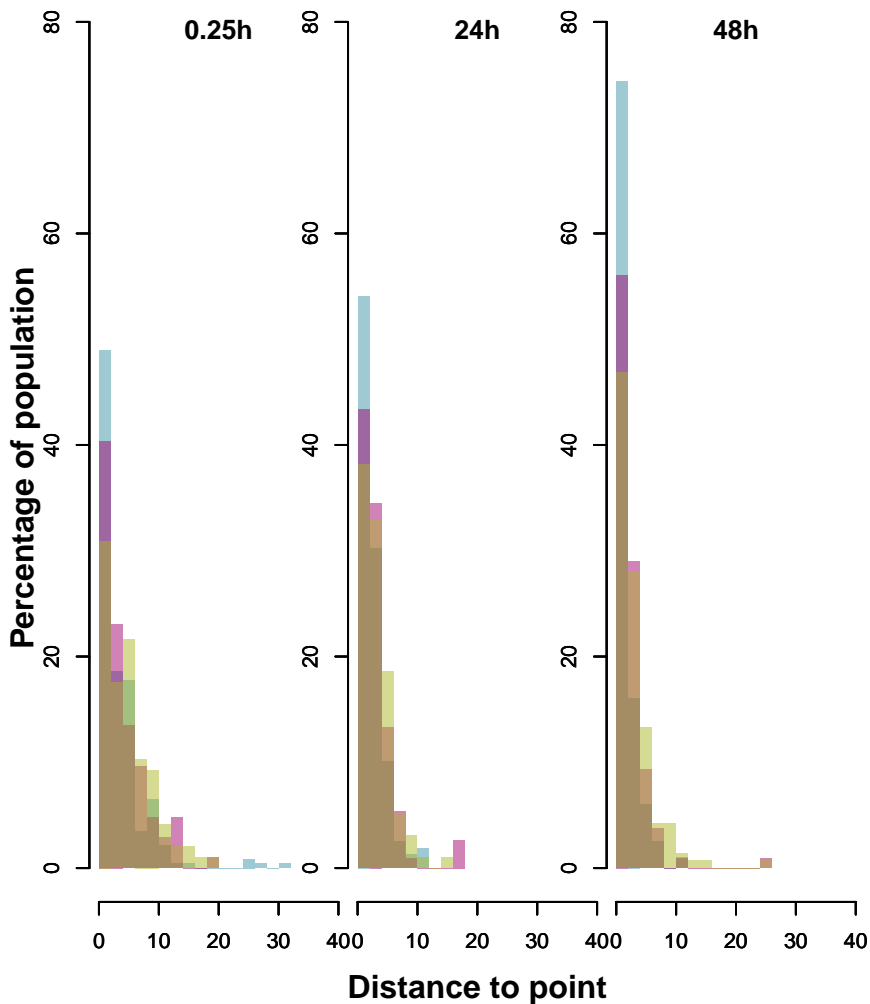

Supplement: Additional file 1: — Figure S1. Histogram showing distribution of distance to point measurements after 0.25, 24 and 48 h. Blue bars represent ancestral populations, green bars represent populations evolved in low resource supply (0.5% FBS) and pink bars represent populations evolved in high resource supply (5.0% FBS). (PDF 5 kb) [file 12862_2017_1117_MOESM1_ESM.pdf]
